# Forever interesting disease: Kawasaki vasculitis – case series

**DOI:** 10.1186/1546-0096-12-S1-P367

**Published:** 2014-09-17

**Authors:** Velma Selmanovic, Aida Omercahic-Dizdarevic, Senka Mesihovic-Dinarevic, Adisa Cengic

**Affiliations:** 1 Allergology, Rheumatology and Clinical Immunology, Children's Hospital Sarajevo, Sarajevo, Bosnia and Herzegovina; 2 Cardiology, Children's Hospital Sarajevo, Sarajevo, Bosnia and Herzegovina; 3 Allergology, Rheumatology and Clinical Immunology, Children's Hospital Sarajevo, Sarajevo, Bosnia and Herzegovina

## Introduction

Kawasaki disease (KD) typically presents in children younger then 5y as a febrile illness with mucocutaneous changes. If untreated, KD can result in coronary aneurisms in 25% patients.

## Objectives

Analysis of 6 patients diagnosed as KD, at Children's Hospital Sarajevo from 2008 to 2014.

## Methods

Retrospective analysis of clinical features, coronary artery abnormalities and treatment outcome.

## Results

Youngest patient was 3 months, oldest 13 years, far more boys 83% (5/6). Seasonal peak was during winter (Jan-Feb) in 50%. Clinicall presentation (figure 2) was consistent with literature. All patient had high inflammatory markers, anemia (patients 1, 2, 5), thrombocitosis. Patient 1. had incomplete KD, with coronary artery aneurisms seen before therapy. Completely responded well to one dose of IVIG. Patient 2.was diagnosed od day 7 of fever; received IVIG, but had unusually prolonged systemic inflammation requesting second IVIG dose and pulses of IVMP plus MTX. Patient 3. is a child with suspected primary immunodeficiency (neutrophil dysfunction in observation). Patient 5. presented with pleuropneumonia requesting active pleural dreinage and had prolonged inflammation. Responded well to second IVIG + IVMP. Patient 6.had splinter haemorragies on his nails.

## Conclusion

KD is diagnosed on clinical basis with supportive laboratory evidence and imaging. Once identified, timely initiation of treatment is imperative in order to quell the inflammatory response and decrease the incidence of long-term sequellae, specifically coronary artery aneurisms. Longitudinall follow-up should be implemented based on risk stratification and individualised to each patient.

## Disclosure of interest

None declared.

